# Photosynthetic acclimation to changing environments

**DOI:** 10.1042/BST20211245

**Published:** 2023-03-09

**Authors:** Armida Gjindali, Giles N. Johnson

**Affiliations:** Department of Earth and Environmental Science, School of Natural Sciences, University of Manchester, Oxford Road, Manchester M13 9PL, U.K.

**Keywords:** abiotic stress, acclimation, environment, light, photosynthesis, temperature

## Abstract

Plants are exposed to environments that fluctuate of timescales varying from seconds to months. Leaves that develop in one set of conditions optimise their metabolism to the conditions experienced, in a process called developmental acclimation. However, when plants experience a sustained change in conditions, existing leaves will also acclimate dynamically to the new conditions. Typically this process takes several days. In this review, we discuss this dynamic acclimation process, focussing on the responses of the photosynthetic apparatus to light and temperature. We briefly discuss the principal changes occurring in the chloroplast, before examining what is known, and not known, about the sensing and signalling processes that underlie acclimation, identifying likely regulators of acclimation.

## Introduction

Growing in continuously changing environments, plants have had to evolve a range of responses to adjust their physiology to the prevailing environmental conditions [1]. Changes can vary from those that last for seconds or minutes, such as light fluctuations caused by passing clouds, to long-lasting ones, such as seasonal temperature oscillations. Plants are equipped with a plethora of mechanisms to cope with transient or sustained changes, adjusting their photosynthesis, primary metabolism, and overall physiology. It is imperative that photosynthetic capacity be fine-tuned to match the downstream metabolism of the plant, to avoid oxidative damage that can occur when light absorption exceeds metabolic capacity [2] and to maximise the efficiency of resource use [3,4]. The process by which plants alter their investment in light-harvesting and -using processes, in response to the environmental conditions they experience, is termed photosynthetic acclimation [5,6].

When electron acceptors downstream of the photosynthetic electron transport chain are limited, energy spillage towards oxygen and the production of harmful Reactive Oxygen Species (ROS) can occur [7]. ROS are continuously produced in the photosynthetic electron transport chain (PETC) and plants have multiple mechanisms to scavenge them [8]. However, if production exceeds scavenging, they can cause oxidative damage to proteins, particularly to the photosystem (PS) II core protein D1 but also under some conditions to PSI, a process termed photoinhibition [9,10]. Photoinhibition lowers photosynthetic capacity, since D1 repair takes hours to be completed [11]. It is therefore important for plants to minimise imbalances between energy absorption and its assimilation through downstream metabolism.

Depending on the duration of environmental change, plants employ different strategies to optimise photosynthesis while shielding the cell from damage. Short-term changes are counteracted by regulatory mechanisms that dissipate excess energy as heat. These are activated and de-activated in seconds to hours following the disturbance in the environmental conditions. In contrast, prolonged environmental changes prompt long-term changes of the leaf proteome, adjusting photosynthesis to the new prevailing conditions. This process of photosynthetic acclimation requires days to be completed and results in a new photosynthetic steady state [12–15]. Along with photosynthesis, wider metabolism exhibits dynamic changes when exposed to an environmental change and a new metabolic state is reached after acclimation is completed [16–19]. However, how different environmental inputs are sensed and how they are translated to signals that initiate a distinct response depending on the environmental stimuli is not yet fully understood. In this review, we will focus on dynamic photosynthetic acclimation to sustained condition changes, briefly identifying the responses involved and then discussing potential sensors that may trigger it.

## Photosynthetic acclimation

When a plant is exposed to a prolonged environmental change, it may adjust its physiology to match the new conditions [5,6,20,21]. Acclimation can be separated into developmental and dynamic processes, depending on the growth stage of the leaf when acclimation occurs [22], with these being somewhat distinct (though overlapping) responses [22,23]. Developing leaves can vary in morphology according to the environmental conditions, with for example leaves developed in high light being thicker, with more cell layers, than those developed in shade. Dynamic acclimation occurs in pre-developed leaves with fixed morphology and involves changes in leaf proteome and metabolome [24,25]. Acclimation gives plants the flexibility to survive in a continuously changing environment, allowing an optimum photosynthetic rate while minimising photo-oxidative damage.

The most obvious environmental stimulus affecting photosynthesis is light and it is the response to this that has been most studied. Both light quantity and quality can vary and it is important for plants to dynamically adjust their photosynthesis depending on each [26].

**Figure 1.**
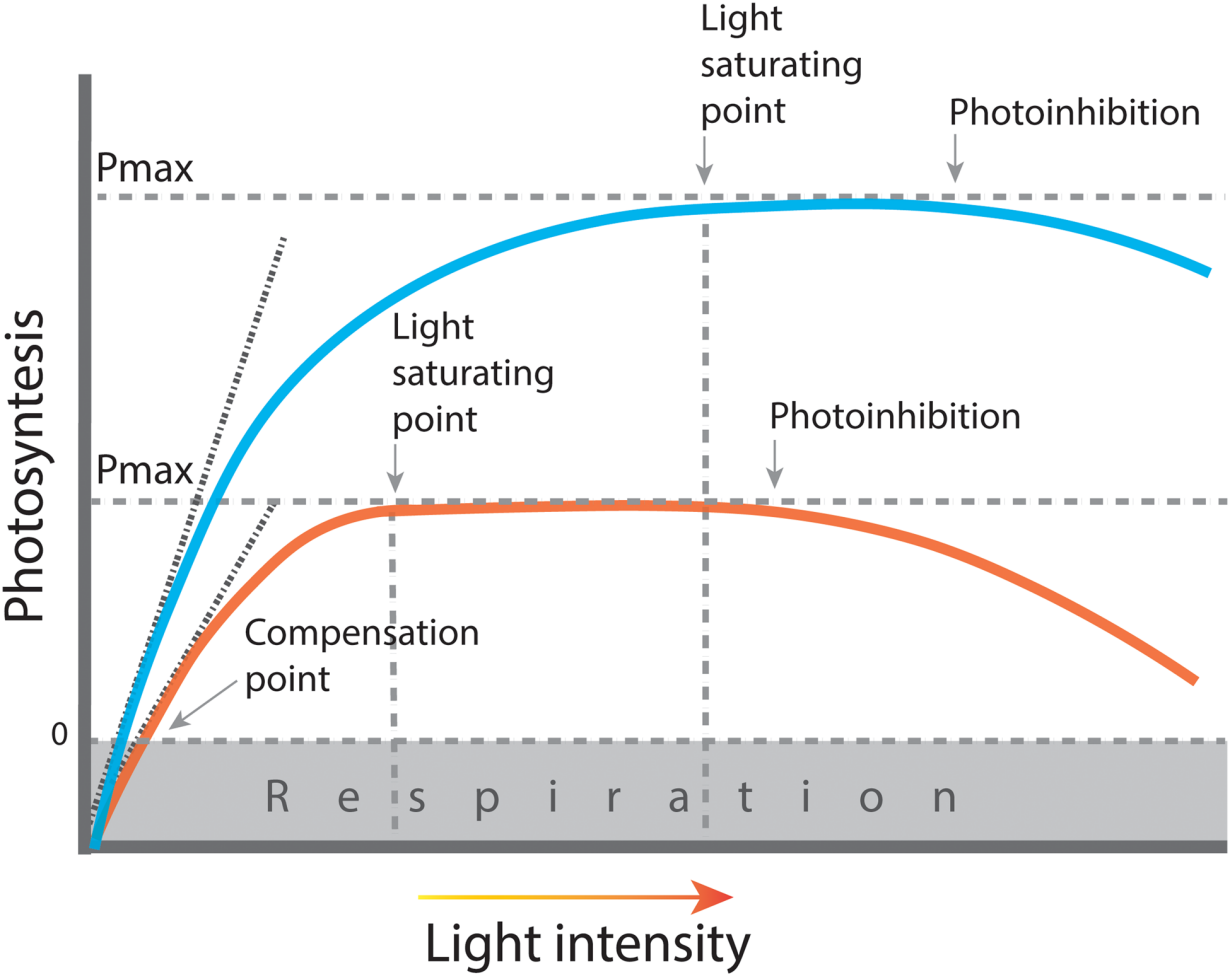
Light response curve of plants grown in low (orange line) and high light (blue line). At low irradiances, photosynthesis increases almost linearly with light intensity. The initial slope of this linear phase corresponds to the maximum quantum efficiency of electron transport through the photosynthetic apparatus while the steepness of the curve as it approaches the light saturating point reflects the rate of use of NADPH by the Calvin–Benson cycle and other metabolic pathways. The light saturation point denotes the light intensity where light stops being the limiting factor. At light saturating point, plants reach maximum photosynthetic capacity. Further increases of light intensity can lead to more energy being absorbed than can be used by the plant, leading to photoinhibition. Plants grown in low light are light saturated at a lower irradiance and reach a lower maximum photosynthetic capacity (Pmax) while plants grown in high light reach a higher maximum photosynthetic capacity are able to use more light before it becomes photoinhibited.

In low light, plants optimise their photosynthetic apparatus, increasing relative investment in chlorophyll antenna and decreasing electron transport capacity, ATP synthase and Calvin–Benson cycle enzymes [27,28]. As a result, low light grown plants have a low maximum photosynthetic capacity (Figure 1). When exposed to high light, plants invest more in electron transport than light capture, increasing cytochrome b_6_f complex, ATP synthase and Rubisco and other enzymes of the Benson–Calvin cycle, resulting in an increase in the overall photosynthetic capacity [29,30]. Decreases in light-harvesting capacity involve changes in the concentration of light harvesting proteins (LCHI and II), which bind chlorophyll a and b, relative to the concentration of reaction centre proteins which only bind chlorophyll a. The chlorophyll a/b ratio is an accessible indicator of the LHC concentration relative to the reaction centres [31,32].

**Figure 2.**
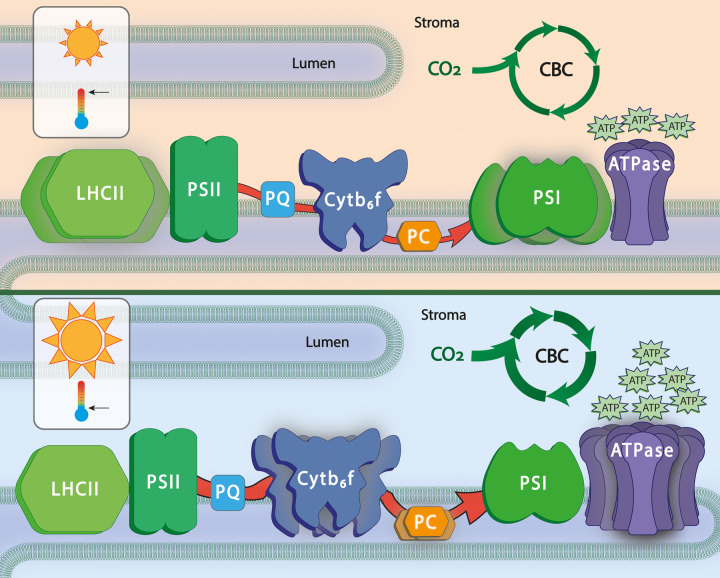
Acclimation to different environmental conditions results in a reshaping of the photosynthetic apparatus. Acclimation to low light and high temperature result in similar changes in the photosynthetic apparatus: increase in light harvesting complex II (LHCII) and photosystem I (PSI) while electron transport and Calvin–Benson cycle (CBC) are reduced. On the other hand acclimation to high light and low temperature lead to increase in the cytochrome b6f complex (Cytb6f), ATP synthase (ATPase) and Rubisco and other enzymes of the CBC and a decrease in the LHCII relative to photosystem II reaction centres (PSII). As a result plants acclimated to high light/low temperature have higher electron transport, CBC and ATP synthesis.

Light acclimation also results in substantial changes beyond what is normally considered to be the core photosynthetic apparatus, with changes across the whole leaf proteome [30]. Photosynthetic acclimation to increased light involves substantial changes in the composition of the photosynthetic apparatus (Figure 2). However transcript levels of genes encoding the two photosystems have been shown to remain largely unaffected by moderately high irradiances [5,22,23,30]. This implies that the composition of the photosynthetic apparatus is regulated post-transcriptionally, i.e. through regulation of translation or protein turnover. Changes in light quality on the other hand, which induce changes in the stoichiometry of PSI and PSII [33,34] lead to distinct gene expression patterns and leaf metabolic states [24]. A time analysis of gene expression alterations ranging from minutes to days revealed different kinetics of gene expression, suggesting that changes in metabolism, downstream of photosynthetic electron flow, could generate feedback signals facilitating acclimation to different light regimes [24].

Although light has a clear link to photosynthesis, other environmental stimuli such as, CO_2_ and changes in ambient temperature affect photosynthesis either directly or indirectly, e.g. by inducing changes in source-sink relationships [5]. For example, a lowering of temperature causes a slowing of electron transport and of enzymes in Calvin–Benson cycle, while energy absorption and electron transfer within complexes are largely unaffected [35,36]. This causes an imbalance between energy trapping and consumption of reductants, posing a threat to the photosynthetic apparatus, with PSII photoinhibition and oxidative stress liable to increase [37]. This is seen as an immediate drop in photosynthetic rate upon transfer to cold, however, if low temperature is sustained over days, photosynthetic acclimation can occur, with photosynthesis at low temperature being restored to levels seen previously at higher temperatures [12]. Such dynamic acclimation to cold involves molecular changes, such as increases in Chl a/b ratio and up-regulation of rate limiting enzymes that in many ways mimic acclimation to high light [38].

Although photosynthetic acclimation is not essential for normal plant growth in controlled environment conditions, there is evidence that it plays an important role in determining the fitness of plants in naturally fluctuating conditions. Athanasiou et al. [22] showed that Arabidopsis plants lacking the chloroplast glucose phosphate/phosphate translocator GPT2, which are deficient in acclimation to increased light, have the same seed yield as wild type when grown in controlled light conditions, but substantially reduced fitness in naturally fluctuating conditions. Similarly, the mutant FUM2, which unable to acclimate to low temperature, has reduced seed yield following episodes of low temperature, compared with the corresponding wild type [39].

## Triggering acclimation: potential sensors

Dynamic acclimation resulting in changing photosynthetic capacity is important for plant fitness in fluctuating environments [5,12,22,40]. However, both the primary sensors of environmental change and the signalling pathways by which those primary sensors give rise to acclimation remain unclear. A change in environmental conditions results in imbalances between energy absorption and metabolic processes. This imbalance is reflected in a change in the redox state of electron carriers, the pH of the thylakoid lumen, ROS levels in the chloroplast, and the concentrations of a wide range of metabolites. There is evidence that each of these may contribute to the environmental sensing preceding acclimation. Section 3 considers some of the candidate sensors that have been identified.

### PQ redox state

Signals originating from the redox state of photosynthesis under different light conditions have been thoroughly studied [41–45]. The oxidation state of the PQ pool is a primary regulator of retrograde signalling affecting the expression of several nuclear genes including PSI subunits under different light qualities in *A. thaliana* [46]. For a thorough review of PQ as sensor and signal initiator see [47]. However, how PQ redox state is translated into signals altering nuclear gene expression is unclear. One of the main sensing hubs proposed to act as the first step in signal transduction from PQ redox state to gene expression is the chloroplast STN7 kinase.

STN7 is activated depending on the PQ redox state and deactivated by the ferredoxin- thioredoxin (Fd-TRX) system and is responsible for phosphorylation of the LHCII and some PSII subunits during state transitions, regulating energy distribution between photosystems at low light [48]. STN7 has been also proposed to function in acclimation, phosphorylating and thus activating unknown signalling molecules [49,50]. One proposed candidate as a retrograde signal is the soluble TSP9 protein. TSP9 is a plant-specific nuclear encoded protein found bound to the thylakoid membrane when not phosphorylated. Upon illumination, it is reversibly phosphorylated by STN7, leading to dissociation from the membrane. This behaviour makes TSP9 a so-far unique candidate for a role in redox-mediated signalling under high light conditions however conclusive evidence for its role is yet to be found [51,52].

### Acidification of thylakoid lumen

Acidification of the thylakoid lumen is an important factor which responds to environmental change. It prompts major changes in energy utilisation, causing protonation of the PSBS protein that in turn results in conformational changes of LHCII and activation of a photoprotective process called non-photochemical quenching (NPQ) [53]. In addition, low lumen pH causes disassociation of Ca^2+^ ions from the Oxygen Evolving complex (OEC) and loss of PSII activity [54]. Most importantly, lumen pH regulates the D1 turnover rate which requires disassembly of the PSII-LHCII super complex and unstacking of grana ultimately affecting electron transport rate and the redox state of the photosynthetic apparatus [55,56]. Lumen pH is also involved in regulation of the cytochrome b/f complex, and so impacts PQ redox state [57]. Acidification has therefore the potential therefore to either directly or indirectly (via NPQ) trigger acclimation responses [58,59]

Lumen acidification has been shown to affect the repair cycle of photo damaged D1 via regulation of proteases involved in its degradation. FtsH's are trans-membrane zinc metalloproteases located on the thylakoid membrane and they are the predominant candidate for degradation of photo damaged D1 protein and protection from photoinhibition [60,61]. A possible mechanism for activation of FtsH kinase is via protonation of amino acid residue of the lumen side of the protein [55]. This causes conformational changes of the monomer, favouring the formation of hexamers which have been shown to be essential for the proteolytic activity [62]. Interestingly, phosphorylation of FtsH hexamers, which enhances the stability of the polymer, is not mediated by STN7 and STN8 kinases in Arabidopsis [63]. This shows that it is ΔpH and not redox state of PQ that regulates FtsH and ultimately D1 repair. Deg1 is another trans-membrane kinase located in the thylakoid membrane and involved in the degradation of damaged D1 and it was also shown to be regulated by lumen acidification [64,65]. In slightly acidic conditions, Deg1 forms hexamers that have higher proteolytic activity than monomers, which are found in neutral or basic pH's. Decreases of lumen pH result in protonation of a His residue on the lumen side of the membrane that initiate polymerisation of Deg1 monomers [64]. Acidification of the lumen therefore acts as an instigator of the repair cycle of D1 preventing decrease in photosynthetic rate caused by photoinhibition. The D1 turnover cycle and the presence of inactive PSII or D1 fragments have been shown to protect PSI from oxidative damage [66] and might therefore function as a sensor or signal to trigger acclimation, especially to differences in light quality altering photosystem ratios [67,68].

### ROS production

Reactive oxygen species (ROS) produced at various steps in the electron transport chain may also act as sensors for a change in environmental conditions and imbalances between energy absorption and assimilation [42,69]. Hydrogen peroxide (H_2_O_2_) can diffuse from the chloroplast creating a cascade of signals ultimately affecting nuclear gene expression, while oxidation of β-carotene by singlet oxygen (^1^O_2_) has been shown to lead to the formation of the volatile compound β-cyclocitral (β-CC) which in turn induces changes in nuclear gene expression [70,71]. Accumulating ROS under excess light conditions promotes the expression of ROS scavenging enzymes such as the cytosolic *ASCORBATE PEROXIDASE 2* (*APX2*) which, interestingly, has also been shown to be regulated by the redox state of PQ in a ROS independent manner [72]. Thus ROS have the potential to act as sensors driving acclimation [73,74]

### Ions

Calcium is a fundamental secondary messenger, with Ca^2+^ signalling playing a role in all eukaryotes [75]. In addition to the cytoplasm and vacuole, Ca^2+^ levels in the chloroplast fluctuate as response to abiotic stress [76,77]. Chloroplasts accumulate Ca^2+^ in a light dependent matter and upon transfer to darkness Ca^2+^ exits the chloroplast within minutes [77,78]. The magnitude of this Ca^2+^ efflux is dependent on both the intensity and duration of light [78]. Stromal Ca^2+^ increases due to several environmental stimuli including temperature changes [77,79], oxidative and salt stress [80,81]. Changes in Ca^2+^ are therefore potential sensors for acclimation.

Stroma levels of free Ca^2+^ are maintained low suggesting that chloroplasts store Ca^2+^ either bound to thylakoid proteins or in an unknown store [77]. Ca^2+^ has been suggested to enter the lumen, possibly through a Ca^+^/H^+^ antiporter [82] where it binds to OEC, as one of its cofactors [54], and to a thylakoid membrane protein CAS (Ca^2+^ sensor) [83]. CAS binds Ca^2+^ in low affinity and high capacity and has been associated with stomata closure as well as tolerance under high light and drought stress in *Arabidopsis* [77,84–86]. Increasing light intensities cause phosphorylation of CAS by STN7/STN8 kinases, whose activity is regulated by the PQ redox state via the Fd-Trx system [83,87–89]. CAS phosphorylation is hypothesised to result in release of bound Ca^2+^ and initiation of signalling cascades such as the inositol 1,4,5-trisphosphate (IP_3_) pathway in *Arabidopsis* integrating cytosolic and chloroplastic Ca^2+^ levels [85,90]. For a detailed review on Ca^2+^ chloroplast signalling see [91].

Potassium ions have also been shown to affect photosynthesis and chloroplast development [92]. K^+^ enters the chloroplast via the KEA1–2 antiporters and the lumen via the K^+^/H^+^ antiporter KEA3 [93,94]. Influx of K^+^ ions in the lumen contributes to the necessary proton motif force (*pmf*) for ATP synthesis while maintaining lumen pH in high enough that it doesn't activate photoprotective mechanisms, allowing higher photosynthetic rate [95,96].

### Metabolites

Soluble sugars are the main products of photosynthesis and play a pivotal role in regulating plant growth and development [97]. The primary photosynthetic export from the chloroplast is triose phosphates (glyceraldehyde-3-phosphate and dihydroxyacetone phosphate) which can be converted to hexose phosphates via the enzyme aldolase. Hexose phosphates in turn act as substrates for sucrose synthesis, which is commonly the sugar exported from the leaf [98]. In the leaf, glucose-6-phosphate (G6P) can be re-introduced in the chloroplast via the glucose-6-phosphate/phosphate translocator GPT2 [99]. GPT2 expression has been shown to rise at high irradiance and affects carbon exchange between chloroplast and cytosol, linking GPT2 with high light acclimation response [23,100].

In sink tissues, hexose sugars, glucose (Glu) and fructose (Fru), are phosphorylated by hexokinases (HXKs) and fructokinases (FRKs) to produce G6P and fructose-6-phosphate (F6P) to be used in downstream metabolism [101,102]. HXKs have been shown to act as sensors of sugar levels but also as signals regulating gene expression in plant cells [101,103]. HXKs can down-regulate photosynthesis, by altering expression levels of photosynthetic genes, and promote tolerance under abiotic stress [104]. HXKs regulate photosynthesis in a sugar mediated manner, affecting stomatal opening [105,106] and water transport by suppressing gene expression levels of aquaporins [107].

Trehalose-6-phosphate (T6P) is another soluble sugar that has been recognised as a sensor for the leaf carbon status and a major signal that regulates sucrose [108,109], lipid [110] and starch synthesis [111] and overall carbon metabolism via interaction of T6P with the SnRK1 protein kinase system [112,113]. The possible role of T6P and trehalose in improving plant growth via regulation of carbon sinks for photosynthates has been review by [114] and [115] respectively.

Sucrose (Suc) can act both as sensor and as signalling molecule during abiotic stress, coordinating gene expression and promoting anthocyanin synthesis [116–118]. Synthesis of Suc from triose phosphates is an important route for recycling phosphate which must be returned to the chloroplast for photosynthesis to occur [119]. Suc has a pivotal role in signalling affecting gene expression regulation via the SNF1 kinase system [117,120].

A significant proportion of carbon fixed in the leaf is transiently stored in the form of starch, synthesised in the chloroplast [121]. Mutants that are impaired in starch synthesis have been seen to have reduced photosynthetic capacity [122,123]. In *Arabidopsis thaliana*, in addition to starch, a large proportion of carbon is accumulated diurnally in the form of the organic acid, fumaric acid [124,125]. Plants lacking the enzyme fumarase 2 (FUM2) are unable to acclimate dynamically to decreases in temperature [12,19,39].

## Signalling between the chloroplast and the nucleus is essential for the acclimation process

**Figure 3.**
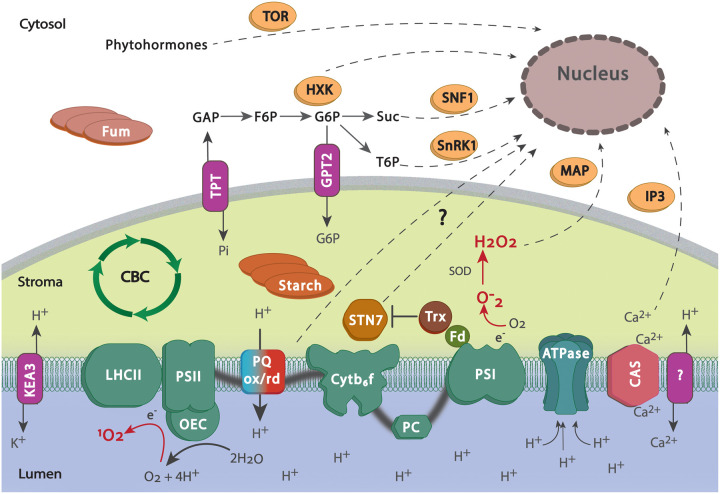
Possible sensors and signals of acclimation. Sensors include acidification of the lumen, the redox state of the plastoquinone (PQ) pool, overproduction of reactive oxygen species (ROS) originating from the photosynthetic chain, the shuffling of ions in the chloroplast lumen and metabolites such as sugars, starch and fumaric acid (Fum). Influx of cations such as potassium and calcium, can maintain a proton motive force (pmf) for ATP synthesis, while ensuring lumen pH doesn't drop too low. Calcium also binds to a transmembrane protein located in the thylakoid membrane, the Calcium Sensor (CAS). CAS has been shown to initiate signalling pathways, such as the inositol 1,4,5-trisphosphate (IP3) pathway, and alter gene expression during different environmental stresses. CAS releases Ca^2+^ upon phosphorylation by the STN7 kinase. STN7 activity is regulated by the PQ redox state through the thioredoxin-ferredoxin system (Trx-Fd). STN7 has been hypothesised to contribute to retrograde signalling phosphorylating an unknown protein that could exit the chloroplast. PQ redox state has been long recognised to regulate the expression of photosynthetic genes, but the mediators of this signalling remain unknown. Reactive oxygen species (ROS) are produced on both sides of the thylakoid membrane and act both as sensor and signalling molecules with hydrogen peroxide (H_2_O_2_) being able to diffuse through the chloroplast membrane and induce changes in gene expression possibly activating the MAP kinases pathway. Glyceraldehyde 3-phosphate (GAP) synthesised in the Calvin–Benson cycle (CBC) is exported to the cytosol through a triose-phosphate transporter (TPT). GAP export is vital for the replenishing of phosphorus stroma pool. In the cytosol, fructose and glucose are phosphorylated by hexokinases (HXKs) and fructokinases (FRKs) to produce glucose-6-phosphate (G6P) and fructose-6-phosphate (F6P). HXKs regulate photosynthetic gene expression in a sugar-dependent manner. G6P can re-enter the chloroplast via the GPT2 transporter where it can be converted and stored as starch. Starch and other carbon sinks, such as fumaric acid (Fum) in the cytosol, act as sensors of the cell sugar level and it are essential for abiotic stress response. Trehalose-6-phosphate (T6P) can be produced from G6P and affect photosynthetic gene expression as well as starch synthesis via the SnRk1 kinase pathway. Sucrose (Suc) acts both as sensor as well as signal coordinating gene expression via the SNF1 kinase pathway. Finally, phytohormones participate in photosynthetic gene regulation during acclimation via the Target of Rapamycin (TOR) pathway.

The photosynthetic apparatus is encoded by genes located both in the chloroplast and in the nucleus. Acclimation of photosynthesis requires co-ordinated changes in gene expression between these genomes, implying that communication between these compartments is essential [126,127]. Signals arising in the chloroplast, controlling nuclear gene expression are termed retrograde signals, those signalling from nucleus to chloroplast are anterograde. Signals coming from the chloroplast (e.g. lumen pH, PQ redox state or ROS production in the chloroplast) would need to signal via retrograde pathways (Figure 3). Metabolic signals may be primarily formed or detected in either chloroplast or cytosol.

ROS are intrinsically unstable, but nevertheless there is evidence that some ROS (e.g. H_2_O_2_) or stable products of ROS metabolism may migrate between compartments [7,69,128]. In addition to direct ROS signalling that involves diffusion of H_2_O_2_ from the chloroplast, signalling cascades mediated by MAP kinases [129,130], and ROS oxidised products have been proposed to signal gene expression changes as a response to abiotic stress [69,131]. Compounds include the isopropanoid precursor methylerythritol cyclodiphosphate (MEcPP) that induces the unfolded protein response in ER [132,133], 3′-phosphoadenosine 5′-phosphate (PAP) that participates in ABA regulated stomata closure [134] and β-cyclocitral (β-CC) that derives from carotenoid oxidation [135].

The role of phytohormones in photosynthetic regulation under optimum conditions has been studied [136–138]. Phytohormones also contribute to regulation of photosynthesis during abiotic stress, protecting it from oxidative damage [139,140]. For example, under drought conditions cytokinins trigger acclimation through alterations of photosynthetic genes encoded in the nuclear via a pathway that involves abscisic acid cross-talk [137,141].

Regulation of photosynthetic gene expression and phytohormones signalling is mediated by the evolutionarily conserved Target of Rapamycin (TOR), a Ser/Thr protein kinase [142]. TOR is a central regulatory hub connecting nutrient, hormonal and stress signals to metabolic and photosynthetic responses [143]. TOR affects the levels of phytohormones and have been shown to be negatively regulated by the SnKR1 kinase as a response to low energy and carbon levels [144,145]. Therefore, phytohormones not only alter photosynthesis but their levels are also regulated by photosynthetic products. The photoprotective role of phytohormones during abiotic stress and their potential role in improving photosynthesis has been recently reviewed by [140] and [146].

Finally, products of photosynthesis, e.g. sugar phosphates may act as retrograde or anterograde signals. As noted above, the transporter GPT2, which exchanges phosphate and sugar phosphates (especially G6P between chloroplast and cytosol) is necessary for dynamic acclimation to light [23,30]. These metabolites may directly signal the metabolic status to control acclimation or may indirectly affect other metabolic processed, leading to control of gene expression.

## Conclusion

The phenomenon of photosynthetic acclimation has been known for over half a century [147] and the physiological and molecular changes involved in light and temperature acclimation are well characterised [3,27,35,148–150]. Nevertheless, we are only now starting to understand the sensing and signalling processes involved. A key unanswered question is how plants integrate environmental fluctuations over time. Many studies have looked at acclimation to different stable conditions, however in nature fluctuations occur rapidly and stochastically [151,152]. Some potential sensors, e.g. PQ redox state and lumen pH, respond directly to the prevailing conditions and therefore fluctuate themselves in line with environmental changes. Accumulation of molecules such as starch, sugars, and organic acids occurs over longer periods. How these different signals are integrated remains unknown. Some progress has been made in understanding the coordination of gene expression between compartments [153–155], but much of the work on retrograde signalling has focused mostly on transcriptional control, whilst there is evidence that translation or protein turnover may be important in the fine-tuning of gene expression that is involved in dynamic acclimation [30]. Most studies to date have looked at acclimation to single environmental parameters whereas there is growing evidence that light, temperature, and CO_2_ availability (mediated by stomatal opening) interact to control photosynthetic acclimation. Understanding these interactions will require the integration of multiple experimental approaches, including multi- ‘omic and modelling approaches.

## Perspectives

- Acclimation of photosynthesis to changes in environmental conditions plays an important role in optimising growth and minimising the negative effects of plant stress.
- Acclimation involves changes in the relative and absolute concentrations of proteins required for photosynthesis, including changes in the investment in light harvesting proteins relative to proteins which capture and use light energy. A range of potential sensors and signals have been identified as being involved in the regulation of acclimation.
- As we learn more about light sensing, challenges remain in trying to understand the integration of different signals and how these are communicated between different cellular compartments and are averaged over time.

## Open Access

Open access for this article was enabled by the participation of University of Manchester in an all-inclusive *Read & Publish* agreement with Portland Press and the Biochemical Society under a transformative agreement with JISC.

## Abbreviations

CAS: Ca^2+^ sensor
CBC: Calvin–Benson cycle
FRKs: fructokinases
FUM2: fumarase 2
Fd-TRX: ferredoxin- thioredoxin
GAP: Glyceraldehyde 3-phosphate
HXKs: hexokinases
IP_3_: 1,4,5-trisphosphate
NPQ non-photochemical quenchingOEC: oxygen evolving complex
ROS: reactive oxygen species
TOR: Target of Rapamycin
